# The use of fluorescence correlation spectroscopy to characterize the molecular mobility of fluorescently labelled G protein-coupled receptors

**DOI:** 10.1042/BST20150285

**Published:** 2016-04-11

**Authors:** Laura E. Kilpatrick, Stephen J. Hill

**Affiliations:** * Cell Signalling Research Group, School of Life Sciences, University of Nottingham, NG7 2UH, U.K.

**Keywords:** diffusion, dimerization, fluorescent ligand, fluorescence correlation spectroscopy, G protein-coupled receptor

## Abstract

The membranes of living cells have been shown to be highly organized into distinct microdomains, which has spatial and temporal consequences for the interaction of membrane bound receptors and their signalling partners as complexes. Fluorescence correlation spectroscopy (FCS) is a technique with single cell sensitivity that sheds light on the molecular dynamics of fluorescently labelled receptors, ligands or signalling complexes within small plasma membrane regions of living cells. This review provides an overview of the use of FCS to probe the real time quantification of the diffusion and concentration of G protein-coupled receptors (GPCRs), primarily to gain insights into ligand–receptor interactions and the molecular composition of signalling complexes. In addition we document the use of photon counting histogram (PCH) analysis to investigate how changes in molecular brightness (*ε*) can be a sensitive indicator of changes in molecular mass of fluorescently labelled moieties.

The plasma membrane of living cells has been shown to be highly structured, with integral membrane proteins and their associated signalling partners organized into distinct microdomains [1,2]. This heterogeneity and compartmentalization adds complexity to identifying specific signalling complexes and is likely to have profound spatial and temporal consequences on intracellular signalling [3]. There is a need to further elucidate the molecular mechanisms that govern signalling within these specific membrane regions. This is particularly pertinent for G protein-coupled receptors (GPCRs) as they are the largest family of proteins in the human genome, numbering over 800 unique members [4], and are integral to a vast range of physiological processes. The membrane localization of GPCRs enables transduction of extracellular signals to the intracellular environment leading to the recruitment of signalling partners (such as G proteins or β-arrestin) and ultimately activation of intracellular signalling cascades. Recent advances have added further complexity to GPCR pharmacology particularly in respect to oligomerization, allosterism and signalling bias [5]. Therefore understanding the molecular consequences of ligand–receptor interactions at the single molecule level as opposed to within whole heterogeneous cell populations would be advantageous.

Fluorescence correlation spectroscopy (FCS) can quantify the real time mobility of fluorescently labelled receptors or ligands within regions of single living cells. To create the confocal detection volume used in FCS, a microscope objective lens with a high numerical aperture is used to focus a laser to a diffraction limited spot (Figure 1a). This use of a pinhole, creates a Gaussian-shaped detection volume approximately 0.25–0.5 fl in diameter (the exact size is dependent on the wavelength of laser excitation used; Figure 1b), which typically encompasses a region of ∼0.3 μm^2^ of plasma membrane containing 1–100 fluorescent particles [6–8]. As fluorescent particles diffuse through they produce time-dependent fluctuations in intensity (Figure 1c). The amplitude of a fluctuation (*δI*) is compared with the mean fluorescent intensity (⟨*I*⟩) at time *t* with that of a subsequent fluctuation at time *t* + *τ*. Using a whole range of *τ* values allows the autocorrelation function (*Gτ*) to be derived. When normalized to the square of ⟨*I*⟩, the autocorrelation can thus be expressed as:

$$G\tau =\frac{\langle \delta I(t)\delta I(t+\tau )\rangle }{{\langle I\rangle }^{2}}$$

**Figure 1:**
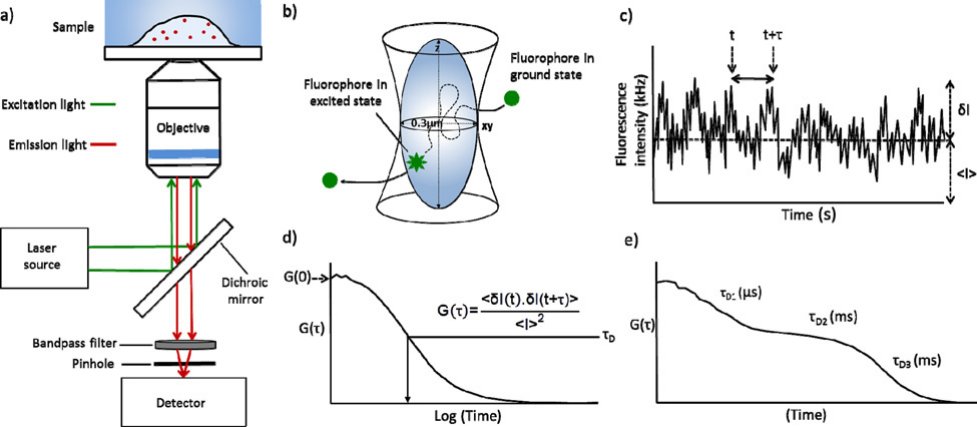
Schematic illustrating the principles of FCS FCS measurements require a confocal microscope fitted with an objective with a high numerical aperture with the basic microscope setup summarized in (**a**). The use of a pinhole creates a Gaussian-shaped detection volume, approximately 0.25–0.5 fl in diameter, and encompassing a plasma membrane region of ∼0.3 μm^2^ (**b**). As fluorescent particles pass through the detection volume, they produce time-dependent fluctuations in fluorescence intensity (**c**). The amplitude of these fluctuations (*δI*) can be compared with that of the mean fluorescence intensity (⟨*I*⟩) at time point *t* with that of a fluctuation at a later time point (*t* + *τ*). Analysis of an ensemble of *τ* values allows the autocorrelation function (*Gτ*) to be determined (**d**) and the average dwell time (*τ*_D_) of the fluorescent species can be derived from the midpoint decay of the autocorrelation curve. A single time-correlated autocorrelation trace can contain multiple components (e.g. *τ*_D1_, *τ*_D2_ etc.; **e**) that represent the different molecular complexes present within the confocal volume distinguished by their different rates of diffusion (typical time scales stated).

The autocorrelation function provides information on the average dwell time (*τ*_D_; obtained from the midpoint of the decay of the autocorrelation curve; Figure 1d) of these fluorescent particles within the confocal volume [7]. This is derived by curve fitting (non-linear) the autocorrelation function using an appropriate biophysical model that accounts for 2D (e.g. within the plane of the membrane) or 3D (e.g. free ligand) diffusion (Figure 1e). *τ*_D_ in conjunction with the calibrated dimensions of the confocal volume allows the average particle number of the respective fluorescent species to be determined. Additionally the diffusion coefficient (*D*) of the lateral mobility of fluorescent moieties within the plasma membrane can be determined using the equation:

$$D=\omega_{0}^{2}/4\tau_{D}$$

where *ω*_0_ is the radial waist of the confocal detection volume.

As the amplitude of the autocorrelation curve is inversely proportional to the concentration of fluorescent particles present within the confocal volume, FCS analysis is most effective when the concentration of fluorescent moieties is low. It is therefore sensitive enough to be used in native tissue samples where receptor expression is typically low [7,8]. However the largest limitation of FCS is that it can only detect mobile fluorescent moieties [8]. Therefore those that are immobile, such as when bound to the cytoskeleton [9], lipid rafts [10] or caveolae [2] will not be detected. A single time-correlated autocorrelation trace can contain multiple components with very different dwell times which are additive (e.g. *τ*_D1_, *τ*_D2_ etc.; Figure 1e). A single trace therefore provides information on the different molecular complexes present within the confocal volume when they can be distinguished by their different rates of diffusion [7]. For example, FCS can separately determine free and receptor bound fluorescent ligands [11]. Typically within a trace, the faster component termed *τ*_D1_ (usually measured in microseconds), represents the photophysics of the fluorophore used or the free diffusion of a fluorescent ligand (if used), whereas more slowly diffusing species (*τ*_D2_, *τ*_D3_) represent molecular complexes (typically measured in milliseconds). FCS has been used by researchers in our laboratory to investigate the diffusion of a range of fluorescently tagged proteins including the Class A GPCRs adenosine A_1_ [12,13], adenosine A_2A_ [13], adenosine A_3_ [1,11], histamine H_1_ [14] and neuropeptide (NPY) Y receptors [15]. FCS has also been used by other research groups to investigate β-adrenoceptors [16], somatostatin receptors [17], type 2 bradykinin [18] and the biogenic amine α_1b_-adrenoceptors, β_2_-adrenoceptors, muscarinic M_1_ and M_3_ and dopamine D_1_ receptors [19]. The diffusion of serotonin 5-hydroxytryptamine 2C (5-HT_2C_) has also been investigated using FCS in both heterologous (HEK293) [20] and native cells (rat choroid plexus epithelial cells) [21].

Although FCS can detect single molecules, it does not truly offer single molecule sensitivity as the parameters derived from the autocorrelation analysis are based on that of an ensemble of readings. Other fluorescence-based techniques can provide complementary measurements to those observed in FCS. Of these, total internal reflection fluorescence microscopy (TIRF-M) can provide true single molecule sensitivity. TIRF relies on the production of an evanescent wave produced when light is totally internally reflected at the boundary of two media with differing refractive indices (e.g. water/glass interface) [22]. The high signal-to-noise ratio of TIRF-M has allowed the mobility of individual fluorescent receptors and ligands to be tracked over timescales of seconds. This has allowed the dynamics and propensity of GPCR oligomerization to be investigated with transient associations and dissociations of muscarinic M_1_ receptors (dimer half-life 0.5 s) [23] and N-formyl peptide receptors [24] identified. Additionally the combined use of TIRF and SNAP labelling has revealed dynamic complex formation of β_1_ or β_2_-adrenoceptors with the propensity of formation differing with subtype and expression level [25]. TIRF-M is therefore more sensitive to determining changes in mass than FCS, however TIRF-M studies are limited to cellular regions close to the interface region due to the lack of depth penetration of the evanescent wave into the sample (∼100–200 nm limit). Fluorescence recovery after photobleaching (FRAP) is another technique with the ability to measure the mobility of fluorescently tagged moieties within the membrane of living cells [26] to shed light on the molecular dynamics of a range of cellular processes. In FRAP, a region of the cell is photobleached using a high laser power, and the recovery of fluorescence is measured and attributed to inward diffusion into this region of unbleached ‘bright’ molecules. Although analysis of data provided by FRAP measurements also provides estimates of diffusion coefficients, FRAP measurements are made over a larger membrane region than those of FCS. Therefore FRAP measurements are likely to be more influenced by the heterogeneity of the membrane region, which may increase the potential impact of limits to free diffusion in the timescales used in FRAP measurements, such as cytoskeleton networks [27]. However unlike FCS, FRAP can estimate the proportion of both mobile and immobile fluorescent particles. Therefore the effect of naturally occurring cellular barriers to diffusion within membranes can be investigated. Measurements derived from FCS and FRAP can therefore complement one another by providing information on diffusion at the micro and macro level respectively.

## Using FCS to provide insights into the molecular pharmacology of GPCRs

As the distinct molecular mechanisms governing the pharmacology of different GPCRs can differ, the sensitivity of FCS is able to detect some of the molecular nuances potentially involved in these processes. For example, the effect of ligand stimulation on diffusion rates can markedly differ between GPCR subtypes. For the adenosine A_1_ receptor, agonist occupancy had no detectable effect on diffusion rates [13], with similar results also observed with 5-HT_2C_ receptors [20], β_2_-adrenoceptors and muscarinic M_1_ receptors [19]. Whereas stimulation of NPY Y1 receptors with the agonist NPY resulted in a significant slowing in their lateral mobility when compared with unstimulated receptors [15]. A similar phenomenon has also been observed for complement C5a tagged with YFP [28].

Two colour cross-correlation spectroscopy (FCCS) is able to separate the emission from two spectrally distinct fluorophores into two detection channels and autocorrelate the fluctuations obtained from both. Cross-correlation analysis of both allows the interaction of both fluorescent species within the confocal volume to be investigated [7]. Filtered FCS (fFCS) is a similar techniques utilizing emission from two channels that can separate auto and cross-correlation functions on the basis of fluorescence lifetime, polarization and spectral properties [29]. Both these techniques have been used to investigate the dynamics of conformational changes of metabotropic glutamate receptors (mGluR) with activation (timescale of transition ∼50–100 μs; [30]). Interestingly the ability of ligand to influence dynamic transitions between active and resting states of the receptor correlated with ligand efficacy.

Changes in diffusion rates with ligand stimulation can also infer interactions of receptors with adaptor proteins such as G proteins or β-arrestin or in the case of NPY Y1 receptors this slowing was believed to be due to interactions with clathrin-coated pits prior to endocytosis effectively immobilizing these receptors for a proportion of their dwell time [15]. These changes may also infer interaction of receptors with each other as higher order structures, such as for bradykinin 2 receptors [18]. However care must be taken in respect to relating changes in diffusion rates derived from FCS to changes in molecular mass, as a 1.6-fold change in *D* will only be seen with an 8-fold change in molecular mass [31].

FCS can also be augmented by the use of other fluorescence-based techniques, such as bimolecular fluorescence complementation (BiFC). In BiFC, a full length fluorescent protein such as YFP or GFP can be split into its corresponding amino and C-terminal fragment [32]. These fragments are themselves non-fluorescent and can be covalently attached to proteins of interest. If these tagged proteins interact with one another the two fragments can refold and reform the full length fluorescent protein. The production of fluorescence is therefore a marker of specific protein–protein interaction. The use of BiFC with FCS has allowed the discrete identification of the diffusion rates of defined molecular complexes, such as adenosine receptors [13] and histamine H_1_ receptor dimers [33], 5-HT_2C_ homodimers [20] and β_2_-adrenoceptors [19]. The irreversible nature of BiFC also allows for the constrainment of precise signalling complexes such as receptors bound to G proteins or adaptor proteins. For example BiFC has been used in FCS to investigate the recruitment of β-arrestin to NPY Y receptors [15]. FCS techniques have also been combined with FRET to investigate the dynamics of conformational changes of syntaxin-1 [34] and calmodulin [35].

## The use of photon counting histogram analysis, to probe changes in the molecular mass of fluorescently labelled species

In respect to measuring changes in molecular mass of signalling complexes, photon counting histogram (PCH) analysis is a more sensitive indicator than autocorrelation analysis, and also provides an alternative measure of fluorescent particle concentration. PCH analyses the same fluorescent fluctuations recorded in the autocorrelation trace, but in respect to their variation in amplitude of fluorescence intensity rather than variation over time. This can yield an estimate of the molecular brightness (*ε*) of the fluorescent species within the confocal volume [36,37]. As molecular brightness is proportional to the number of fluorescent particles within a molecular complex, changes in *ε* can more accurately illustrate changes in mass. For example the formation of a GPCR dimer should theoretically be represented by a doubling in molecular brightness when compared with monomeric controls (assuming 1:1 stoichiometry of protein to fluorescent label). PCH analysis has indicated the formation of GPCR dimers of 5-HT_2c_ [20,21] muscarinic M_1_ and M_2_, α_1b_-adrenoceptors, β_2_-adrenoceptors and dopamine D_1_ [19], and has also been used to characterize epidermal growth factor receptor [38], nuclear retinoid X receptor [39] and dynamin 2 [40] dimers. Additionally PCH analysis can also probe the symmetrical mode of recruitment to GPCRs of adaptor proteins, such as β-arrestin2 to NPY Y1 receptors [15]. Autocorrelation and PCH analysis are therefore complimentary to one another, in that they can provide information of the molecular composition and mobility of fluorescent complexes.

## The use of fluorescent ligands in FCS

The relatively recent development and use of fluorescent ligand technologies has allowed the complex nature of GPCR pharmacology to be further elucidated, particularly in respect to ligand binding, allosterism and dimerization [5,41–43]. It is worth noting that considerations are needed when using fluorescent ligands in respect to the pharmacophore chosen, the length of the chemical linker and fluorophore used, as there is the potential that any one of these factors may confer changes to pharmacology when compared with the unmodified parent ligand [41]. The use of fluorescent ligands in FCS is advantageous due to the profound difference in molecular mass, and therefore the diffusion characteristics, of free and receptor bound ligand which can be easily deconvolved by autocorrelation analysis [7]. Fluorescent ligands freely diffuse in three dimensions within the confocal volume with a typical dwell time between 50 and 100 μs (*τ*_D1_ [7]), however upon interaction with membrane bound receptors (which can only diffuse in two dimensions) there is a substantial slowing in ligand diffusion. These profound differences in diffusion rates, allow the molecular composition of signalling complexes to be defined. To date, we have used fluorescent ligands in conjunction with FCS to characterize the adenosine A1 [12,44], adenosine A_3_ [1,11] and histamine H_1_ receptors [14], whereas other research groups have used the same approach to investigate β_2_-adrenoceptors [16], galanin [45] and somatostatin receptors [46]. In all FCS studies utilizing fluorescent ligands, ligand/receptor complexes have been found to exist as two distinct components within the autocorrelation trace (termed *τ*_D2_ and *τ*_D3_) with discrete rates of diffusion (typically 1–20 ms for *τ*_D2_ and 10–700 ms for *τ*_D3_ respectively) implying that multiple states or populations of receptors may exist at any one time.

GPCRs have been proposed to exist in a range of ligand specific conformational states [47]. The diffusion characteristics of the *τ*_D3_ component observed in fluorescent ligand-based FCS has been suggested to reflect this, as its characteristics are sensitive to the nature of the fluorescent ligand used during FCS experiments. The use of either a fluorescent antagonist (CA200645; [11]) or agonist (ABEA-X-BY630; [1]) can selectively label the inactive (R) or high affinity active (R*) forms of the adenosine A_3_ receptor. Under certain conditions *τ*_D2_ can reflect the duration of ligand occupancy at the level of the receptor [11]. For example, if the fluorescent ligand dissociates from the receptor during its transit through the confocal volume then an apparently quicker diffusion time will be recorded by the autocorrelation analysis. This is most noticeable when a fluorescent ligand identifies both *τ*_D2_ and *τ*_D3_ components, whereas the fluorescently tagged receptor records only the slower component [11]. In this situation, allosteric ligands can dramatically reduce the residence time of the fluorescent ligand on the receptor and effectively increase the number of particles with a fast *τ*_D2_ diffusion coefficient [11]. It is also worth stressing, however, that both *τ*_D2_ and *τ*_D3_ may not simply represent distinct receptor states, but may be a composite of the diffusion coefficients of multiple ligand–receptor complexes [7]. Notwithstanding these complexities, FCS in conjunction with fluorescent ligand technology represents a powerful and sensitive technique to begin to tease out ligand specific receptor states at a single receptor resolution within a potentially heterogeneous population.

## Abbreviations

BiFC: bimolecular fluorescence complementation
FCS: fluorescence correlation spectroscopy
FRAP: fluorescence recovery after photobleaching
FRET: fluorescence resonance energy transfer
GPCR: G protein-coupled receptor
5-HT_2C_: serotonin 5-hydroxytryptamine 2C
NPY: neuropeptide Y
PCH: photon counting histogram
TIRF-M: total internal reflection fluorescence microscopy
